# The Effects of Different Dietary Patterns on Bone Health

**DOI:** 10.3390/nu16142289

**Published:** 2024-07-17

**Authors:** Xiaohua Liu, Yangming Wu, Samuel Bennett, Jun Zou, Jiake Xu, Lingli Zhang

**Affiliations:** 1 School of Exercise and Health, Shanghai University of Sport, Shanghai 200438, China; liuxiaohua202204@163.com (X.L.);; 2 School of Biomedical Sciences, The University of Western Australia, Perth, WA 6009, Australia; 3 Shenzhen Institute of Advanced Technology, Chinese Academy of Sciences, Shenzhen 518055, China; 4 School of Athletic Performance, Shanghai University of Sport, Shanghai 200438, China

**Keywords:** dietary patterns, intermittent fasting, caloric restriction, vegetarian diets, high-fat and high-sugar diet, high-protein diet, bone health

## Abstract

Bone metabolism is a process in which osteoclasts continuously clear old bone and osteoblasts form osteoid and mineralization within basic multicellular units, which are in a dynamic balance. The process of bone metabolism is affected by many factors, including diet. Reasonable dietary patterns play a vital role in the prevention and treatment of bone-related diseases. In recent years, dietary patterns have changed dramatically. With the continuous improvement in the quality of life, high amounts of sugar, fat and protein have become a part of people’s daily diets. However, people have gradually realized the importance of a healthy diet, intermittent fasting, calorie restriction, a vegetarian diet, and moderate exercise. Although these dietary patterns have traditionally been considered healthy, their true impact on bone health are still unclear. Studies have found that caloric restriction and a vegetarian diet can reduce bone mass, the negative impact of a high-sugar and high-fat dietary (HSFD) pattern on bone health is far greater than the positive impact of the mechanical load, and the relationship between a high-protein diet (HPD) and bone health remains controversial. Calcium, vitamin D, and dairy products play an important role in preventing bone loss. In this article, we further explore the relationship between different dietary patterns and bone health, and provide a reference for how to choose the appropriate dietary pattern in the future and for how to prevent bone loss caused by long-term poor dietary patterns in children, adolescents, and the elderly. In addition, this review provides dietary references for the clinical treatment of bone-related diseases and suggests that health policy makers should consider dietary measures to prevent and treat bone loss.

## 1. Introduction

Bone is one of the most important organs in the human body and plays a variety of roles, such as support, protection, and hematopoiesis. Bone is a complex and active organ. Under normal circumstances, bone formation and bone resorption are in a dynamic balance. If the balance is broken, it will cause bone loss and eventually lead to osteoporosis (OP) [1]. At present, the incidence of osteoporosis is gradually rising, and is affected by many factors, including genetics, age, diet, hormone levels, and lifestyle [2,3].

Bone health is closely related to the body’s intake of the nutrients in food, such as proteins, inorganic salts, vitamins, etc. [4,5]. The relationship between nutrients and bone health has received widespread attention, but only considering the impact of a single nutrient on bone health is not comprehensive. The daily diet is a mixture of various nutrients that interact with each other and have an impact on bone health. Taking into account the impact of different foods, and adjusting dietary patterns may be a better choice for preventing OP [2]. This article discusses dietary patterns by searching for the keywords “dietary patterns” and “bone” on Pubmed. The database, which is current up to 2024, includes relevant animal and clinical studies that have evaluated the effects of common dietary patterns on bone health, such as bone mineral content, bone strength, bone metabolism indicators, and fracture risk. The results show that there have been significant changes in lifestyle and dietary structures in the past few decades. High-sugar, high-fat, and high-protein diets have been widely adopted due to their abundance and availability. High-calorie diets have become widespread and an important reason for obesity [6]. Obesity increases the risk of diabetes, hypertension, cardiovascular disease, and other chronic diseases. Previous studies showed that obesity has a positive effect on bones, but this view has been overturned in recent years. Fortunately, people are gradually realizing the adverse effects of a high-sugar and high-fat diet pattern. At present, intermittent fasting, caloric restriction, and a vegetarian diet, combined with moderate exercise, are widely respected as strategies with which to reduce weight and improve metabolism, but their impact on bone health is not clear [7]. Studies have shown that a caloric restriction diet has adverse effects on bone health, but this is still controversial, and the effects of an intermittent fasting diet and a vegetarian diet are still unclear [8,9]. Therefore, this paper discusses five dietary patterns—intermittent fasting, caloric restriction, vegetarian diet, HFSD, and HPD—to clarify their potential relationship with bone health. It is revealed that the timely supplementation with appropriate amounts of calcium, vitamin D, and dairy products can effectively prevent OP, providing a reference for how to choose the appropriate diet in the future, and how to prevent bone loss in children, adolescents, and the elderly due to long-term unhealthy diets.

## 2. Intermittent Fasting and Bone Health

### 2.1. Classification of Intermittent Fasting

Intermittent fasting refers to the intermittent reduction in caloric intake or fasting from food or drink for different periods of time, which can range from a few hours to a full 24 h [10]. The first appearance of this term was in religion, where religious believers fasted during Ramadan or Yom Kippur [11]. Later, as this method of fasting was found to be effective in reducing body weight, intermittent fasting began to appear widely in people’s daily lives and was considered a healthy way of eating. It should be noted that there is no one definitive fasting program for intermittent fasting, which is divided into four main types depending on the duration and degree of fasting—alternate-day fasting, modified fasting program, restricted feeding, and Ramadan fasting [12]—and their specific fasting protocols are shown in Table 1.

### 2.2. Basic Research on Intermittent Fasting and Bone Metabolism

Intermittent fasting protocols are considered an appropriate strategy with which to improve various inflammatory and lifestyle-related diseases [13,14]. The most natural intermittent fasting behavior that can be observed in nature occurs during the hibernation of black bears, brown bears, and polar bears. During hibernation, the level of osteoblast activity in the skeletons of these animals decreases dramatically [15], but, in addition to the fasting factor, it is also likely to be caused by the reduced mechanical stimulation of the skeleton by the reduced physical activity of the hibernating animals. Hisatomi et al. [16] found that the width of the lumbar vertebral body and cortical bone thickness in fasted rats tended to decrease compared with those in an ad libitum group, and the bone mineral density (BMD) of the lumbar vertebral body in the fasted group was significantly lower than that in an ad libitum group; the minimum cross-sectional moment indicating “flexural strength” and the polar moment indicating “torsional strength” in the fasted group were lower than those in the non-fasted rats, which also had a negative effect on the bone strength of the rats. Although Hisatomi et al. [16] showed that fasting may be detrimental to BMD and bone strength, the longer duration of fasting in their study was more of a simulation of the effects of prolonged fasting on bone for extreme conditions without food. Majed et al. [17] found that the serum levels of the bone formation biomarkers osteoprotegerin (OPG), alkaline phosphatase (ALP), and osteocalcin (OCN) were significantly increased, and the bone resorption markers tartrate-resistant acid phosphatase (TRAP)-5b, amino-terminal cross-linking telopeptide of type I collagen (NTX-1), and deoxypyridinoline (DPD) were significantly decreased in rats with glucocorticoid-induced osteoporosis subjected to intermittent fasting for 16–18 h per day for 90 days, suggesting that intermittent fasting slows the progression of glucocorticoid-induced osteoporosis by inhibiting osteoclast activity and promoting osteoblast osteogenesis. Similarly, intermittent fasting was found to resist the decrease in BMD caused by a ketogenic diet, and to reduce the increase in the serum bone resorption marker TRAP caused by a ketogenic diet [18]. Both studies suggested that, to some extent, intermittent fasting can counteract the negative effects of disease or diet on bone health, but the effects of different intermittent fasting regimens on bone health may not be the same, and because experimental animals do not have fixed dietary durations, it is difficult to determine the equivalent duration of intermittent fasting in animal models and humans, and to date, animal models investigating the effects of intermittent fasting on bone are not available (Table 2).

### 2.3. Clinical Research on Intermittent Fasting and Bone Metabolism

A scholar who collected blood samples from 23 individuals during their normal lives and during the fasting period (Ramadan) found that Ramadan fasting reduced blood parathyroid hormone (PTH) concentrations, but did not have a significant effect on the blood markers of bone metabolism [19]. Studies have shown that the persistent overproduction of the PTH leads to enhanced bone resorption [20], so Ramadan fasting is thought to be potentially beneficial for bone conversion. Interestingly, most patients with rheumatoid arthritis or spondyloarthritis have found their symptoms relieved after fasting during Ramadan, which may be related to the downregulation of the expression of the pro-inflammatory factors c-reactive protein (CRP), interleukin-1β (IL1β), and interleukin-6 (IL6) [21]. Rodopaios et al. [22] measured serum 25-hydroxyvitamin D concentrations and BMD in 200 adults who adhered to religious fasting for decades and 200 non-fasters and assessed their dietary vitamin D intake and sunlight exposure and found that the 25-hydroxyvitamin D concentrations were lower in the fasters than in the non-fasters in winter and spring, but the BMD did not differ between the two groups; thus, Nikolaos et al. concluded that long-term intermittent fasting did not affect bone health differently. In addition to religiously related Ramadan fasting, the effects of alternate-day fasting regimens and caloric restriction on bone metabolic markers in overweight and obese people were explored in a 6-month randomized controlled trial, which showed good weight reduction in the alternate-day fasting group as well as in the caloric restriction group, and no significant effects on the bone mineral content (BMC), BMD, or the bone metabolism-related markers type I collagen carboxy-terminal peptide (CTX-1) and OPG [23]. David et al. [24] showed similar results and found no effect of 24 h fasting on serum CTX-1, procollagen type I N-terminal propeptide (PINP), or PTH. Martens et al. [25] also showed that 6 weeks of time-restricted feeding in middle-aged and elderly non-obese people showed no difference in the total BMD or regional BMD from that of a control group, and that 6 weeks of time-restricted feeding did not result in a reduction in bone mass in middle-aged and elderly people.

It can be seen from the above research that the effect of intermittent fasting on bone health is still inconclusive, and different results have been obtained from human experiments and animal experiments. Animal experiments have shown that intermittent fasting can resist bone health damage caused by drugs or diet; no effect on BMD or bone microstructure has been found in human studies, and the serum markers of bone turnover are inconsistent due to differences in intermittent fasting regimens (Table 3).

## 3. Caloric Restriction and Bone Health

Caloric restriction is a dietary strategy that reduces or restricts the intake of calories from food while maintaining the body’s nutritional needs. This dietary strategy has been shown to benefit a variety of tissues and organs in the body [26]. At first, caloric restriction was considered to be a healthy and long-lived diet, but it was later found to have a significant effect on weight improvement [27]. Studies have found that caloric restriction has a significant impact on inflammation, insulin resistance, and cardiovascular disease, all known risk factors for osteoporosis and fractures [28,29]. Therefore, exploring the effects of caloric restriction on bone health is also a hot research topic.

Caloric restriction significantly reduces body weight and improves body composition, so many studies related to caloric restriction and bone health have focused on bone marrow fat. Jay et al. [30] found that caloric restriction led to elevated serum TRAP and decreased insulin-like growth factor 1 (IGF-1) and OCN concentrations, which negatively affected bone structure. Exercise reduced serum TRAP concentrations and attenuated the deterioration of bone microarchitecture due to the HFD and caloric restriction. In another study with the same results as those of Jay’s study, Cody et al. [31] found an increase in the bone marrow fat content of the femoral epiphysis after caloric restriction combined with an exercise intervention in mice, an increase in femoral cortical porosity, and a decrease in cortical bone thickness and cortical bone volume fraction, thus suggesting that exercise during caloric restriction may have a negative effect on bone. Some scholars conducted caloric restriction experiments on growing mice for 3 and 9 weeks, and found that serum leptin decreased by 52% and 88%, IGF-1 decreased by 33% and 39%, body size decreased, BMD and BV/TV decreased significantly, Tb.Sp increased significantly, and the bone marrow fat content also increased significantly [32]. Furthermore, aging itself causes an increase in bone marrow fat [33] and, therefore, caloric restriction may exacerbate the effects of bone marrow fat on aging bones. Based on this, Duque et al. [34] studied long-term caloric restriction in aging rats for 12 months, and the rats in the caloric restriction group also showed significantly increased levels of bone marrow adiposity, in addition to significantly decreased serum osteocalcin levels, tibial BMD, and BV/TV compared to rats fed ad libitum during the same period. From the above studies, it is clear that the effect of caloric restriction on bone marrow adiposity is different from that on adiposity in other parts of the body, which may be explained by the increased lipid differentiation of bone marrow mesenchymal stem cells (BMSCs) from bone-to-bone differentiation due to an insufficient energy supply, and the enhanced lipogenic differentiation of BMSCs may result from a decrease in serum leptin due to caloric restriction. This was also verified by Devlin et al. [35], who found that a daily leptin treatment improved the increase in bone marrow adiposity due to an HFD or caloric restriction, thereby protecting bone health.

Caloric restriction promotes bone marrow adipogenesis, which is only one aspect of bone health, but also affects bone mass and bone microarchitecture. Talbott et al. [36] suggested that caloric restriction induces a decrease in estrogen and insulin levels in aged rats, both of which may act together to reduce osteoblast activity relative to osteoclast activity, resulting in reduced bone density and strength. Marko et al. [37] found that caloric restriction led to further reductions in BMD and cortical bone thickness in the femur and tibia of aging mice, and that voluntary running exercise did not counteract the negative effects of caloric restriction on bone, in general agreement with the previous findings of Cody [31]. LaMothe et al. [38] indicated that caloric restriction results in a significant reduction in tibial length, total cross-sectional area, and cortical thickness in aged rats compared to naturally aged mice of the same age. Behrendt et al. [39] similarly showed that caloric restriction results in a significant reduction in femoral BMD, cortical thickness, and fracture strength. A similar phenomenon was found when caloric restriction was studied in estrogen-deficient de-ovulated mice. After 8 weeks of caloric restriction, the de-ovulated mice showed reduced BMD, a reduced trabecular number, increased trabecular separation, and reduced cortical bone thickness and bone volume [40]. The above study reveals that caloric restriction has a greater effect on cortical bone, which is in general agreement with a report by Mark et al., who found that caloric restriction mainly affects cortical bone and does not affect cancellous bone [41]. It has been shown that exercise combined with caloric restriction can lead to a reduction in bone strength [42], and this reduction in bone strength is most likely due to the negative effects of caloric restriction on cortical bone. However, not all scholars who have conducted studies on caloric restriction and bone health have concluded that it negatively affects bone. For instance, Jeon et al. [43] found that short-term caloric restriction may not affect bone mass or bone metabolism in type 2 diabetic rats, and Fontinele et al. [44] showed that caloric restriction minimizes the effects of aging on the medial condyle of the femoroacetabular joint. In contrast, Villareal et al. [45] observed that calorie restriction, although somewhat affecting BMD in middle-aged and elderly subjects, did not cause impaired bone quality or increased bone turnover, and that bone loss may occur early after the onset of caloric restriction, but then the body develops compensatory mechanisms to prevent an increase in the rate of bone turnover, which in turn may preserve bone quality. The reason why these studies have not produced completely consistent results may be due to the different disease models for which caloric restriction was studied, or the different age stages of the animals. In addition, there are differences in the protocols for caloric restriction in animal models, such as the degree of caloric restriction and the duration of the restriction.

Many people interested in fitness and weight loss, and athletes, use caloric restriction to control weight and decrease body fat. Dennis et al. [46] found that the reduction in body weight caused by caloric restriction was accompanied by a significant reduction in the BMD of the total hip, greater trochanter of the femur, and spine. This team also investigated the effects of caloric restriction on bone metabolism and BMD in non-obese young adults. Two years of caloric restriction resulted in a significantly lower BMD of the femoral neck, total hip, and lumbar spine in the non-obese adults compared to the casual fasting group, and a significant increase in serum TRAP and a significant decrease in bone-specific alkaline phosphatase (BSAP) one year after the caloric restriction intervention [47]. Razny et al. [48] found that 3 months of caloric restriction was associated with a significant increase in the serum bone resorption marker CTX-1 in conjunction with a reduction in body weight. On the other hand, Yasuda et al. [49] noted that cumulative bone resorption effects, as well as a slight DNA damage response, were detected in female collegiate judo athletes after weight reduction by caloric restriction prior to competition. In addition, it was found that an adequate nutritional intake (vitamins and minerals) during caloric restriction did not cause changes in the serum bone turnover markers CTX-1 and BSAP, although it caused a decrease in the BMD of the lumbar spine and hip region [45]. In contrast, Villareal et al. [50] conducted a calorie restriction, as well as an exercise combined with diet, intervention program in 107 obese older adults, and found that caloric restriction resulted in a significant reduction in the total hip BMD in older adults, while the exercise combined with diet program intervention reduced this effect.

Caloric restriction has had inconsistent effects on bone in different animal models or populations, and long-term caloric restriction may affect cortical bone, leading to an increased incidence of fractures. For the aging population, caloric restriction may inhibit primary aging (reduced oxidative stress, DNA damage, core body temperature, etc.) and secondary aging (reduced inflammation, obesity, etc.) [51], thus potentially improving the effects of oxidative stress and DNA damage on bone remodeling balance during aging. However, the available evidence suggests that caloric restriction is mostly negative for bone [52], so it is important to consider future implementation options, including the degree and duration of caloric restriction, in order to achieve positive effects on the bones of older adults.

## 4. Vegetarian Diet and Bone Health

Vegetarian diets are better known than intermittent fasting and caloric restriction, and are a common dietary strategy for fitness and weight loss. Vegetarian diets are divided into three main categories based on the degree of dietary restriction: vegan diets (diets that do not contain any animal products), lacto-vegetarians (consuming dairy products but not eggs), and lacto-ovo-vegetarians (consuming dairy products and eggs) [53]. Because vegetarian diets typically contain less saturated fat and cholesterol, and because of the health-promoting effects of increased dietary fiber, vegetarians typically have lower total serum cholesterol and LDL cholesterol levels and lower blood pressure, and may have a reduced incidence of obesity, diabetes, hypertension, metabolic syndrome, ischemic heart disease, cardiovascular disease, and certain cancers [54]. Although there are many benefits to a vegetarian diet, many scholars are concerned about whether a long-term vegetarian diet may lead to inadequate nutritional intake and, thus, have an impact on the bone health of the body.

Since the dietary habits and characteristics of experimental animals are different from those of humans, it is not meaningful to investigate the effects of vegetarian diets on bone health through vegetarian interventions on the diets of experimental animals, so studies investigating the effects of vegetarian diets on bone health are mainly randomized controlled experiments on vegetarians and people on general diets. Lau et al. [55] noted that older female vegetarians had a significantly lower hip BMD compared to omnivores, and that the BMD of vegetarians appeared to be positively correlated with energy, protein, and calcium intake. A previous meta-analysis showed that vegetarians had lower femoral neck and lumbar spine BMDs than omnivores [56], and this was confirmed by a subsequent meta-analysis including 20 studies and 37,134 subjects, which found that vegans had a higher risk of fracture than omnivores [57]. It has been suggested that the reduced BMD and increased fracture risk of vegetarians may be due to a general deficiency of vitamin B12 among vegetarians, which can lead to reduced IGF-1 synthesis, and thus affect bone health [58]. However, some studies have also shown that vegetarian diets do not affect bone health. Chuang et al. [59] noted no significant differences in changes in the BMD or bone trabeculae scores over a three-year interval between vegetarian and non-vegetarian women aged 65–90 years. Knurick et al. [60] compared the BMD of three groups of normal adults who consumed meat, lacto-vegetarian, and vegan diets, and found that those who consumed lacto-vegetarian and vegan diets had an approximately 30% less protein intake than those who consumed meat, but it did not affect their BMD. And Tesar et al. [61] also found no differences in the cortical bone and trabecular bone density, nor in the urinary creatinine and Ca concentrations of lacto-ovo-vegetarians compared to non-vegetarian postmenopausal women. Therefore, it is also believed that although vegetarians reduce their intake of animal-derived protein, the reduced intake of animal-derived protein is compensated for by the intake of protein from plants and legumes, and thus does not affect bone health [62]. Brants et al. [63] indicated that older vegetarian populations are within the guidelines regarding a healthy diet in terms of protein, fat, and carbohydrate percentages compared to omnivorous populations, and that vegetarian diets were more nutrient dense than omnivorous diets. In addition, related studies [64] have shown that increased plant-based food intake in healthy middle-aged populations with normal BMDs can be good for improving BMC due to the wide variety of micronutrients and phytochemicals contained within plant-based diets. This may be related to the presence of high amounts of antioxidants in plant-based diets [65], such as carotenoids, which alleviate aging-induced oxidative stress and delay bone loss in older adults [66]. According to a recent study, a vegetarian diet characterized by a high intake of potassium-rich nutrients (such as fruits and vegetables) was associated with a low dietary acid load, which is associated with lower bone resorption, thus promoting bone health [67]. This mechanism may also be a possible reason why vegetarians can protect their bone health despite the lack of protein and nutrients they consume compared to omnivores.

**Figure 1:**
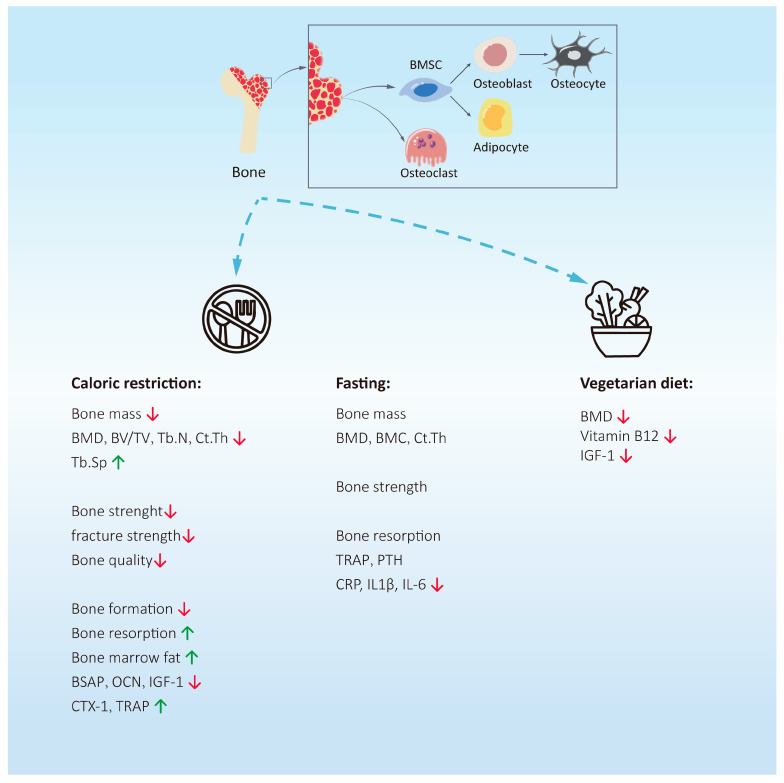
The effects of calorie restriction, intermittent fasting, and vegetarian diets on bone health. Caloric restriction can reduce bone mass and bone strength, inhibit bone formation, and promote bone resorption. The effect of fasting on bone mass is not clear. Interestingly, it can effectively reduce the expression of pro-inflammatory factors. Studies have shown that vegetarian diets may reduce bone mass by reducing the synthesis of vitamin B12 and IGF-1.Abbreviations: BMD = bone mineral density; BSAP = bone-specific alkaline phosphatase; OCN = osteocalcin; IGF-1 = insulin-like growth factor 1; CTX-1 = type I collagen carboxy-terminal peptide; TRAP = tartrate-resistant acid phosphatase; CRP = c-reactive protein; IL1β = interleukin-1β; IL6 = interleukin-6; IGF-1 = insulin-like growth factor 1. ↑ = The expression level of this substance is upregulated. ↓ = The expression level of this substance is downregulated.

Intermittent fasting, calorie restriction, and vegetarianism are common dietary strategies to limit energy intake and are highly favored by people interested in weight loss. Intermittent fasting includes fasting during Ramadan, improved fasting plans, and fasting every other day. Heat limitation also includes varying degrees of heat limitation. Vegetarianism includes a vegan diet, lacto-vegetarian diet, egg–milk vegetarian diet, etc. Due to different dietary strategies, the daily intake of nutrients varies, and the impact on bones is also inconsistent. In addition, we found that the research on animal models is uneven and not completely consistent with the results of human studies, and further research is needed to clarify these differences (Figure 1).

## 5. High-Sugar and/or High-Fat Dietary Patterns and Bone Health

People with obesity are highly susceptible to cardiovascular disease, stroke, and diseases such as diabetes mellitus and OP, the incidence of which is gradually increasing and has become a public health problem worldwide [68,69]. Previous scholars used to consider obesity to be a protective factor for bones because greater body weight increases mechanical loads to promote bone health, but as research has progressed, it has been found that obesity leads to a dramatic increase in the prevalence of OP, fractures, and other conditions. Obesity is inextricably linked to a high-sugar diet (HSD), HFD, and HSFD patterns. An HSFD can cause metabolic disorders, inflammatory responses, and affect the hippocampus, leading to impaired memory function [70,71,72]. Nutrients such as fat, sugar, and protein play a major role in bone metabolism and the maintenance of bone health, and the relationship between an HSFD and bone health has attracted extensive attention from scholars.

### 5.1. HSD and Bone Health

#### 5.1.1. Effect of HSD on Bone Mass and Bone Strength

Western diets contain a large amount of carbohydrates, including sucrose, fructose, and glucose, and sugar intake has been shown to have a negative impact on bone mineral balance [73]. Sugar can negatively affect bone metabolism by affecting calcium homeostasis. The effect of an excessive intake of sugar-sweetened beverages (SSBs) on bone health has become the focus of research. Bone mass increased rapidly during childhood and adolescence, which is the key period of peak bone mass accumulation. A meta-analysis in 2021 showed that there was a significant negative correlation between SSB intake and forearm BMC or whole-body BMD in children and adolescents. In adults, female SSB intake is negatively correlated with BMD, but has no significant effect on males [74]. Bragança et al. [75] analyzed the relationship between the intake of SSBs and bone health in 6620 Brazilian young people aged 18–23, and found that the intake of SSBs was related to the BMD of the lower lumbar spine, but not to the BMD of the total body. This may be because the subjects of this study were young people. If the high intake of SSBs continues, it may lead to the decline of BMD in the total body. A long-term cohort study found that a sustained high intake of SSBs in adolescence and early adulthood was associated with fat weight gain, but not with BMC at the age of 20 [76]. Therefore, public health initiatives for the consumption of SSBs in children and adolescence are still of practical significance.

Animal studies have found that the bone strength of weaned female and male rats significantly decreased after 5 weeks on a high-sucrose diet (43 g/100 g), and the bone weight, and calcium and phosphorus concentrations in the tibia decreased more significantly in females [77]. Bass et al. [78] found that rats on a high-fructose (40% fructose, 10% glucose) diet had higher bone mass and better bone microarchitecture, with a higher BV/TV, Tb.Th, and lower BS.BV in the distal femur, and that the tibia was also able to withstand a greater maximal bending load when compared to a 12-week high-glucose (50% glucose) diet, suggesting that a high-glucose diet compared to a high-fructose diet has a more pronounced negative effect on bone health.

#### 5.1.2. Effect of HSD on Bone Metabolism

Glucose feeding for 8 weeks resulted in a lower femoral and tibial BMD, reduced ALP and total phosphorus in the bone, and decreased calcium intake in adolescent female rats, suggesting that glucose could have more deleterious effects on mineral homeostasis and bone than fructose [79]. Felice et al. [80] constructed a rat model for metabolic syndrome (MS) using a fructose-enriched diet. Significant changes were found in the femoral bone microstructure of rats with metabolic syndrome, with a 20% reduction in osteoclast density and a 30% reduction in the osteoclast-covered (TRAP-positive) bone surface of cancellous bone. These changes may have an effect on the lipogenic/osteogenic differentiation of BMSCs by regulating the Runx2/PPARγ (runt-related transcription factor 2/peroxisome proliferator-activated receptors-γ) ratio, which in turn affects the process of bone remodeling.

In conclusion, an HSD may differentially affect bone mass, bone strength, the bone microenvironment, and bone mineralization. A long-term HSD may modulate the lipogenic/osteogenic differentiation of BMSCs, increase the risk of future metabolic disorders in the body and, consequently, adversely affect bone health.

### 5.2. HFD and Bone Health

#### 5.2.1. Effect of HFD on Bone Mass and Bone Strength

The effect of an HFD on bone health has always been a widely debated topic, and many researchers have come to different conclusions about the impact of an HFD on bones. Traditionally, an HFD has been considered to increase bone mass and promote bone health, as mechanical loads applied to bones have a positive impact [81]. Male adolescents whose obesity is caused by an HFD have greater muscle area and a higher BMD [82]. Kim et al. [83] evaluated the bone parameters of 12–19-year-old adolescents, and found that bone mass was increased in obese adolescents, suggesting that obesity has a positive impact on bone health. However, with the gradual deepening of research, it is now believed that the main factors affecting BMD and BMC are weight, lean weight (skeletal muscle), and mechanical stimulation. The acquisition of bone mass is mainly during the growth period; children and adolescents are in the important stages of bone growth. The ages 1–4 and adolescence are the two stages of rapid bone mass growth, typically reaching peak bone mass around 23. Children and adolescents are in the golden period of physical growth and development. The rapid growth of BMD in these two stages is related to a rapid increase in body weight. Obesity is often accompanied by high body weight. A rapid increase in body weight increases the mechanical load of bones. In addition, an increase in muscle area leads to more tendon–bone connections at the bone, and an increase in muscle strength leads to greater traction, resulting in rapid bone growth. Leonard et al. [84] compared the BMD of the tibia and radius in obese and normal-weight adolescents and found that the BMD of the tibia in obese adolescents increased significantly. Also, in those with obesity, load-bearing bones (lumbar spine, hip, femur) change more than non-load-bearing bones (upper limbs) [85].

Since the load-bearing parts of rats are different from those of human beings, the weight-bearing forelimb accounts for about 40% of its body weight, while the lumbar spine does not bear weight, so the changes in the bone mass of the femur and tibia are more obvious than those of the lumbar spine, and similar results have also been observed in mice [85,86]. However, with an increasing incidence of bone-related diseases, an HFD is considered to be a risk factor for bone loss. The maturation of mouse bones is completed around 3 months; at 6 weeks of age, and after 10 weeks of an HFD, weight gain and bone loss were observed. However, at the same age of 16 weeks, in mice fed an HFD for 10 weeks, bone mass was not affected, indicating that obesity has a negative impact on early bone development [87]. Tang et al. [88] found that mice with HFD-induced obesity had reduced BMD, Tb.N, Tb.Th, and BV/TV, increased Tb.Sp, and showed significant deterioration of bone trabeculae. A meta-analysis showed that a decrease in bone mass and bone strength was detected in the femur and tibia of HFD-fed mice [89]. However, Doucette et al. [90] found that an HFD (60% kcal) increased the volume of marrow adipose tissue in mice, but had no adverse effects on bone remodeling.

#### 5.2.2. Effect of HFD on Bone Metabolism

The impact of an HFD on bone formation is influenced by multiple factors. Leptin can inhibit the differentiation of BMSCs into adipocytes and promote bone differentiation. Liu et al. [91] found that serum leptin levels are higher in obese children, accompanied by high BMD. Postmenopausal obese women have higher levels of leptin, which are positively correlated with BMI [92]. In addition, the protective effect of leptin on bones is similar to that of estrogen, acting on the receptor activator of the NF-κB/receptor activator of the NF-κB ligand/osteoprotegerin (RANK/RANKL/OPG) signaling pathway which downregulates RANKL expression, upregulates OPG expression, and promotes osteoblast differentiation. However, other studies have shown that leptin can inhibit bone formation through the central nervous system and promote osteoclast differentiation and bone resorption [93]. Therefore, the effect of high levels of leptin on bone metabolism in obese patients needs further study. Consistently, obese postmenopausal women have been found to have decreased levels of the bone formation marker PINP and decreased osteoblast synthesis [94], and decreased OCN levels in obese patients [95]. In comparison, TNF-α is significantly elevated in obese patients, and TNF-α has been found to inhibit osteoblast proliferation by downregulating Runx2 and osterix (OSX) expression [96,97]. In addition, vitamin D levels are deficient in obese people [98]. Vitamin D promotes osteogenesis and decreases bone resorption by increasing the intestinal absorption of calcium and phosphorus, the renal reabsorption of calcium and phosphorus, and decreasing parathyroidal (PTH) synthesis.

In animal experiments, obese mice showed increased bone resorption and were prone to bone loss [96]. Both a short-term and long-term HFD significantly increased the bone resorption marker CTX, and led to impaired bone mineral density and bone microstructure in obese mice [99]. Obese rats had increased osteoclast proliferation and differentiation because of the upregulation of the expression of RANK and RANKL [100]. Studies have found that the bone resorption marker cathepsin K (CTSK) is highly expressed in the adipose tissue of obese patients and animals [101]. After 12 weeks of HFD feeding, the differentiation of osteoclasts in mice was increased. Even though the osteogenic differentiation of BMSCs in obese mice was increased, with detectable levels of osteoblast transcription factors Runx2 and OSX and bone formation marker OCN in the femur, bone loss still occurred, indicating that osteoclast-mediated bone resorption plays a leading role in the effect of obesity on bone [102]. The changes in adipose tissue secretory proteins caused by obesity also affect bone resorption. Oshima et al. [103] found that adiponectin promotes the proliferation and differentiation of osteoblasts, but also inhibits the bone resorption of osteoclasts. The level of RANKL in adiponectin^−/−^ mice decreases, while the level of OPG increases, thereby inhibiting the formation of osteoclasts [104].

The mechanisms by which an HFD affects bone also involve the induction of inflammatory gene expression and osteoclastogenesis [105]. The BMD was significantly decreased in HFD-fed (40% fat ratio) rats after 10 weeks, and the immunohistochemical data showed an increased expression of the inflammatory factors TNF-α, IL-6, and PPAR-γ, and a decreased expression of adiponectin (APN) [106]. An HFD was found to induce the expression of several pro-inflammatory cytokines (including TNF-α, IL-1, and IL-6) which could activate osteoclasts and increase bone resorption via the RANKL/RANK/OPG axis in the bone marrow microenvironment, resulting in altered bone mass, reduced bone mass, impaired bone regeneration, and increased BMAT volume in HFD-fed (60% kcal) rats [107]. Consistently, HFD-fed rats also showed increased production of IL-1, IL-6, and TNF-α, increased NF-κB, and decreased PPAR-γ expression in BMSCs [108]. Bone mass was reduced in mice after 3 weeks of an HFD treatment, with increased osteoclast formation and bone resorption activity, as well as increased expression of osteoclastogenesis regulators (RANKL, TNF-α, and PPAR-γ) in mice following 6- and 12-week HFD treatments [102]. Other studies have also found a significant increase in TNF-α, IL-6, and PPAR-γ; a decrease in adiponectin; and a decrease in osteoblast differentiation with severe bone loss in HFD-fed obese rats [106]. Interestingly, there is also a high level of resistin in the serum of obese patients and in obese mice [109]. Resistin could activate the NF-κB signaling pathway and increase the expression levels of inflammatory factors such as TNF-α and IL-6, thereby promoting osteoclast differentiation. In addition to inhibiting bone formation, inflammatory cytokines TNF-α can also promote the expression of the macrophage colony-stimulating factor (M-CSF) and RANKL-inducing factor, and promote osteoclast differentiation. IL-6 overexpression in transgenic mice could lead to severe damage of the trabecular bone and cortical bone [110]. Furthermore, an HFD could affect oxidative stress of mice. Xiao et al. [111] used an HFD to feed 4-week-old mice for 13 weeks, and the HFD group showed an increase in total antioxidant capacity and antioxidant enzyme activity, while their GSH/GSSG (glutathione/oxidized glutathione) ratio was significantly reduced, their malondialdehyde was dramatically increased, and ROS accumulated, ultimately leading to the dysregulation of bone metabolism. Zengin et al. [112] investigated the effects of a low-carbohydrate HFD to determine whether sex-specific effects on bone health existed, and showed that a 4-week HFD led to a decrease in bone mass in male rats, whereas there was no significant effect in female rats, suggesting a gender difference in the effects of an HFD on bone health.

### 5.3. HSFD and Bone Health

#### 5.3.1. Effect of HSFD on Bone Mass and Bone Strength

An HSFD can lead to obesity and diabetes, two interrelated diseases also related to OP. An HSFD significantly decreased BMD in ovariectomized (OVX) rats [113]. A long- term HSFD has had significant adverse effects on bone morphology and mechanics in rats [114]. Tian et al. [115] fed an HFD, HSD, and standard diet to male C57bL/6J mice aged 6–7 weeks for 8, 16, and 24 weeks. The results showed that after short-term feeding, both the HFD and HSD showed positive effects on bone mass, while after long-term feeding, the bone mass of the HFD mice decreased, and the bone mass of the HSD mice increased first and then decreased, confirming that an HSD and HFD have different regulatory effects on bone mass. Another study reached a different conclusion, that is, that a long-term high-fat intake led to a decrease in bone mass and an increase in the structure model index (SMI) in BALB/cByJ mice, while an HSD did not affect bone health. The difference between the two studies may be due to the difference in the mouse models used and the feeding age [116].

#### 5.3.2. Effect of HSFD on Bone Metabolism

The effect of an HSFD on bone metabolism has received extensive attention. After 10 weeks of an HSFD, the cortical thickness, cross-sectional area, and maximum load of 9-week-old female C57bL/6J mice were significantly reduced, the amounts of TRAP and RANKL in the serum were increased, and the OCN and OPG/RANKL had no significant changes [117]. An HSFD significantly decreased the BMD, serum OCN content, urine DPD content, and the osteoclast-specific expression gene CTSK in ovariectomized (OVX) rats, suggesting that an HSFD shows high bone absorption, effecting in bone health [113]. Studies have shown that the harmful effects of an HSFD may be more related to changes in cortical bone [118]. Most studies have suggested that the bone loss caused by an HSFD may be due to the increased expression of inflammatory factors and the increased activity of osteoclasts.

There are more studies on the effects of an HSFD on bones, but no uniform conclusions have been reached. The effects of an HSFD on bone mass and bone microstructure are multifaceted, which may be related to the breed of experimental mice, their age, sex, type of feed, and duration of feeding. Overall, an HFD is detrimental to bone. In addition, unlike humans, large mice walk on all fours and the femur is subjected to less weight-bearing and mechanical stimulation. Therefore, further experimental approaches need to be developed and optimized to avoid this difference. The exact mechanism by which an HSFD causes an increase in adipose tissue, which not only affects the development of the skeletal muscular system, but also brings about a decrease in physical activity that can lead to bone loss, is complex, and future directions may focus on the molecular mechanisms behind the effect.

## 6. High-Protein Dietary Patterns and Bone Health

Proteins are one of the components of the body and play an important role in promoting growth and development. Due to its ability to effectively control weight, reduce appetite, and increase muscle mass, a high-protein diet has received widespread attention. In the case of a sufficient supply of calcium and vitamin D, protein intake can effectively prevent the occurrence of osteoporosis. At present, the definition of a high-protein diet (HPD) has not yet been determined, and a recommended daily protein intake of 0.83 g/kg is recommended. However, most regulations require a protein intake threshold between 1.2 and 2.0 g/kg per day. A protein intake exceeding 1.5 g/kg is considered a high-protein diet (HPD) [119]. The adverse effects of a HPD on kidney disease patients have been confirmed, but the impact on bone health is not yet clear.

**Figure 2:**
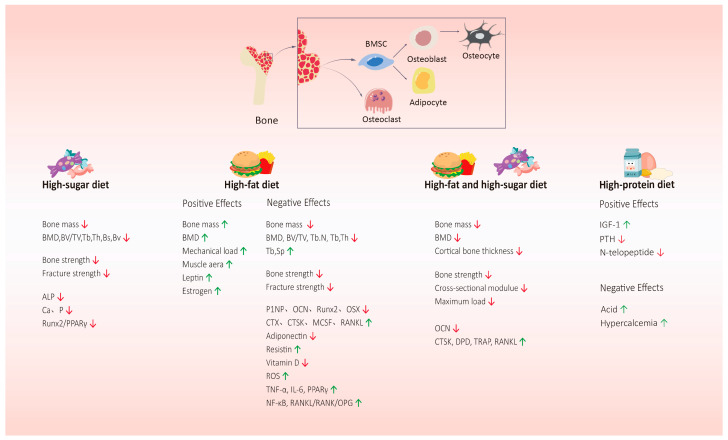
The effects of a high-sugar diet, high-fat diet, high-fat and high-sugar diet, and high-protein dietary patterns on bone health. A high-sugar diet, high-fat diet, and high-fat and high-sugar diet can reduce bone mass and bone strength, inhibit bone formation, and promote bone absorption. But research shows that a high-fat diet also plays a positive role in bone health. The relationship between a high-protein diet and bone health is still controversial. It plays an active role in bone health by upregulating IGF-1, inhibiting the PTH, and promoting the intestinal absorption of calcium, while its hypercalciuria and other effects may have adverse effects on bone. Abbreviations: ALP = alkaline phosphatase; Runx2/PPARγ = runt-related transcription factor 2/peroxisome proliferator-activated receptors-γ; BMD = bone mineral density; PINP = procollagen type I N-terminal propeptide; OCN = osteocalcin; OSX = osterix; CTX-1 = type I collagen carboxy-terminal peptide; MCSF = macrophage colony-stimulating factor; TRAP = tartrate-resistant acid phosphatase; IGF-1 = insulin-like growth factor 1; PTH = parathyroid hormone; IL6 = interleukin-6. ↑ = The expression level of this substance is upregulated. ↓ = The expression level of this substance is downregulated.

Protein is 22% of the composition of bone. The amino acids and peptides in proteins are conducive to the absorption of calcium. Adequate dietary protein is the premise upon which to ensure bone remodeling. Studies have shown that protein intake has a positive effect on bone [120]. Most epidemiological studies have shown that there is a significant positive correlation between a long-term high-protein intake and BMD. A meta-analysis in 2017 showed that an HPD had a protective effect on lumbar BMD, but had no effect on the total hip, femoral neck, or whole-body BMD or bone biomarkers [121]. Another study found that after an HPD (2.2 g/kg/d), there was no significant change in BMC or BMD (i.e., whole body and lumbar spine) [122]. An HPD was found to increase the intestinal absorption of calcium, increase the production of IGF-1, inhibit the PTH, and improve muscle strength and quality, which are beneficial to bone health [123]. By studying the effects of low- (0.7 g/kg), medium- (1.0 g/kg), and high- (2.1 g/kg) protein diets on bone health, it was found that compared with the medium-protein diet, urinary calcium under the low-protein diet was significantly reduced, whereas urinary calcium under the HPD was significantly increased, and the PTH under the HPD was significantly reduced [124]. Studies have also shown that IGF-1 can regulate bone remodeling by promoting osteoblast activity and reducing bone resorption [125]. Dawson-Hughes et al. [126] showed that increasing protein intake from 0.78 to 1.6 g/kg per day could lead to an increased serum IGF-I level, and a lower bone resorption index with a decreased level of n-telopeptides in urine. The relationship between an HPD and bone health remains controversial. By giving different levels of protein diets to Wistar rats for 3 weeks, an HPD was found to have little effect on bone metabolism-related indicators [127]. Other studies have shown that medium- and high-protein diets and exercise groups could increase BMD and affect cortical bone, but would not cause changes in bone turnover markers in obese rats [128]. An HPD may produce more acids during the process of protein metabolism, while the kidneys cannot completely neutralize the remaining acid load, which might have adverse effects on bones [129]. Further research is needed to clarify the relationship between an HPD and bone health (Figure 2).

## 7. Intake of Calcium, Vitamin D, and Dairy Products and Bone Health

Healthy adults contain about 1 kg of calcium, 99% of which is deposited in the bones and teeth, and only 1% of calcium exists in the blood, extracellular fluid, and soft tissue [130]. Calcium plays a key role in bone mineralization and maintaining intracellular and extracellular homeostasis. Calcium is an essential element. The body can only obtain it from food, including dairy products, fish, beans, vegetables, and fruits. Yao et al. [131] showed that higher calcium intake was positively correlated with lumbar BMD, but interestingly, this relationship was more obvious in women. In a cohort study, the long-term intake of a sufficient amount of calcium in children increases BMD and reduces the risk of osteopenia [132]. Ma et al. [133] randomly divided 220 Han adolescents aged 12–14 years old into a low-calcium group (300 mg/d), medium-calcium group (600 mg/d), and high-calcium group (900 mg/d). After one year of this intervention, their bone mineral density was determined. The results showed that compared with the low-calcium group, the percentage of the femoral neck BMC in the medium- and high-calcium groups increased significantly, suggesting that an increase in calcium intake contributes to an increase in bone mass in adolescents. Studies have shown that drinking water rich in calcium can effectively improve bone metabolism in men and women [134]. In addition, calcium homeostasis is also regulated by vitamin D. The combination of vitamin D and dietary calcium supplements can effectively reduce the incidence of fractures [135,136]. Liu et al. [137] also concluded that dairy products containing calcium and vitamin D have beneficial effects on bone mineral density and can prevent the occurrence of osteoporosis in postmenopausal women. However, a meta-analysis in 2015 showed that dietary calcium intake had nothing to do with fracture risk, and that there was no clinical trial evidence that increasing dietary calcium intake could prevent fractures [138].

During the growth process, dairy products provide about 50–60% of calcium intake and 20–30% of protein intake [139]. The intake of dairy products is beneficial at all ages, especially for children and adolescents [140,141]. Li et al. [142] discussed the changes in bone health of Chinese healthy children aged 4–6 years old during the year of supplementing with dairy products. The results showed that compared with the control group, the BMD and BMC of the left forearm of the children in the dairy group were significantly higher, while at the sixth month, the serum 25 (oh) D and IGF-1 levels in the dairy group increased, and the PTH was inhibited, but there was no significant difference after one year. The daily intake of milk containing 250 mg of calcium can effectively prevent BMD loss of the hip and femoral neck in Chinese postmenopausal women [143]. A large sample size study in Switzerland showed that compared with women who drink a cup (200 mL) of milk every day, women who drink three or more cups of milk every day are more likely to have fractures, and the fracture risk of the latter is 16% higher than that of the former, while the fracture risk of men is lower than that of women [144]. At the same time, the author also found that a large amount of milk intake is related to inflammation and oxidative stress, as milk contains a large amount of D-galactose, which increases the risk of fractures and mortality [144]. Therefore, the proper intake of dairy products can play a positive role in bone health.

Therefore, an adequate intake of calcium, vitamin D, and dairy products can effectively improve the peak bone mass of adolescents, prevent bone loss in postmenopausal women, and prevent the occurrence of senile osteoporosis.

## 8. Conclusions

OP is one of the more common skeletal system diseases, and its incidence is increasing year by year. The latest meta-analysis found that the incidence of OP was the highest in Asia, at 24.3%, much higher than that in Europe and the Americas [145]. The prevention of OP mainly includes two aspects, namely, increasing the peak bone mass in adolescence and delaying or preventing bone loss in old age. Dietary patterns play an important role in affecting bone health. Our results show that the relationship between intermittent fasting and bone health needs more research to confirm that caloric restriction and vegetarian diets can reduce bone mass. The negative impact of an HSFD on bone health is far greater than the positive impact of the mechanical load. The relationship between an HPD and bone health remains controversial. Calcium, vitamins, and dairy products play an important role in preventing bone loss. The diet of European and American countries contains high protein and high fat (such as cheese, meat, hamburger, French fries, etc.), which can easily cause obesity and affect bone health [2]. In the Asian population, the diet pattern of eating more fruits, vegetables, and soybeans is related to a reduction in fracture risk and the risk of osteoporosis [146]. Studies have shown that the recommended dietary calcium intake is 800–1200 mg per day. The recommended daily phosphorus intake for adults is 700 mg, and that for adolescents during growth is 1250 mg. The recommended intake of protein is 0.8 g/kg per day, which is increased to 1.0–1.2 g/kg per day for the elderly [5]. Due to different dietary habits in different regions, most of the results of this study are from European and American states, and the results will be different, which will also be considered in the future. It is important to supplement calcium, vitamins, and dairy products in time to improve bone mass. It is recommended that a large amount of fruits, vegetables, low-fat dairy products, whole grains, poultry, fish, nuts, and beans are consumed, as they have been proven to have a positive impact on bone health.

Promoting a healthy diet is very important to reduce the incidence of OP. However, the molecular mechanism between different dietary patterns and bone health is still unclear, which needs further study. A reasonable dietary composition, good dietary habits and methods, and exercise are the basis for ensuring human health. It is important to conduct health promotion during childhood and adolescence. Calcium, vitamin D, and dairy intake are effective for preventing age-related OP. It is necessary for health policy makers to consider measures to prevent and treat osteoporosis in the elderly. Further understanding the relationship and molecular mechanisms between dietary patterns and bone health will pave the way for designing better dietary structures to improve bone health.

## Figures and Tables

**Table 1:** Major intermittent fasting protocols.

	Protocol
Alternate-Day Fasting	Alternate fasting days and eating days.
Modified Fasting Regimens	Fasting days allow for 20–25% of daily energy requirements, alternating with feeding days.
Time-Restricted Feeding	Consume random energy intake at specific times of the day and prohibit the intake of calorie-containing foods and beverages at specific times.
Ramadan Fasting	Followers of some religions must fast from sunrise to sunset during Ramadan.

**Table 2:** Application of different intermittent fasting protocols in animal models.

Types of Intermittent Fasting	Subjects	Effects on Bone	Reference
96 h of single fasting	6-week-old male Wister rats	Lumbar vertebra BMD ↓ Bone strength index minimum cross-sectional moment, polar moment ↓	[16]
16–18 h of fasting per day	Rats with glucocorticoid-induced osteoporosis	Serum OPG, ALP, OCN ↑ TRAP-5b, NTX-1, DPD ↓	[17]
Alternate-day fasting	6-week-old male Sprague Dawley rats + ketogenic diet	BMD ↑ Serum TRAP ↓	[18]

Abbreviations: BMD = bone mineral density; OPG = osteoprotegerin; ALP = alkaline phosphatase; OCN = osteocalcin; TRAP = tartrate-resistant acid phosphatase; NTX-1 = amino-terminal cross-linking telopeptide of type I collagen; DPD = deoxypyridinoline. ↑ = The expression level of this substance is upregulated. ↓ = The expression level of this substance is downregulated.

**Table 3:** Application of different intermittent fasting protocols in human studies.

Types of Intermittent Fasting	Gender (M/F)	Age (Year)	Subjects	Effects on Bone	Reference
Ramadan fasting	18/5	18–42	Saudi Arabian Muslims	Serum PTH during Ramadan fasting ↓.	[19]
Ramadan fasting	143/257	24–58	People with and without Ramadan fasting habits	25-hydroxyvitamin D concentrations were higher in winter and spring fasters than in non-fasters ↓.	[22]
Alternate-day fasting	8/54	18–65	Overweight and obese people	BMC, BMD, serum CTX-1, OPG showed no significant difference.	[23]
24 h of single fasting	8/8	19–25	Healthy subjects	Serum CTX-1, P1NP, PTH showed no significant difference.	[24]
8 h food restriction	10/12	55–79	Healthy elderly subjects	No significant effect on BMD.	[25]

Abbreviations: PTH = parathyroid hormone; BMD = bone mineral density; BMC = bone mineral content; CTX-1 = type I collagen carboxy-terminal peptide; OPG = osteoprotegerin; PINP = procollagen type I N-terminal propeptide. ↓ = The expression level of this substance is downregulated.

## References

[1] Raggatt L.J., Partridge N.C. (2010). Cellular and molecular mechanisms of bone remodeling. J. Biol. Chem..

[2] Muñoz-Garach A., García-Fontana B., Muñoz-Torres M. (2020). Nutrients and Dietary Patterns Related to Osteoporosis. Nutrients.

[3] Cheng C.H., Chen L.R., Chen K.H. (2022). Osteoporosis Due to Hormone Imbalance: An Overview of the Effects of Estrogen Deficiency and Glucocorticoid Overuse on Bone Turnover. Int. J. Mol. Sci..

[4] Tsugawa N., Shiraki M. (2020). Vitamin K Nutrition and Bone Health. Nutrients.

[5] Rizzoli R., Biver E., Brennan-Speranza T.C. (2021). Nutritional intake and bone health. Lancet Diabetes Endocrinol..

[6] Ji T., Fang B., Wu F., Liu Y., Cheng L., Li Y., Wang R., Zhu L. (2023). Diet Change Improves Obesity and Lipid Deposition in High-Fat Diet-Induced Mice. Nutrients.

[7] Waddington G.S. (2021). COVID-19, mental health and physical activity. J. Sci. Med. Sport.

[8] Peng Y., Zhong Z., Huang C., Wang W. (2023). The effects of popular diets on bone health in the past decade: A narrative review. Front. Endocrinol..

[9] Ogilvie A.R., McGuire B.D., Meng L., Shapses S.A. (2022). Fracture Risk in Vegetarians and Vegans: The Role of Diet and Metabolic Factors. Curr. Osteoporos. Rep..

[10] Grajower M.M., Horne B.D. (2019). Clinical Management of Intermittent Fasting in Patients with Diabetes Mellitus. Nutrients.

[11] Klempel M.C., Kroeger C.M., Bhutani S., Trepanowski J.F., Varady K.A. (2012). Intermittent fasting combined with calorie restriction is effective for weight loss and cardio-protection in obese women. Nutr. J..

[12] Patterson R.E., Laughlin G.A., LaCroix A.Z., Hartman S.J., Natarajan L., Senger C.M., Martinez M.E., Villasenor A., Sears D.D., Marinac C.R. (2015). Intermittent Fasting and Human Metabolic Health. J. Acad. Nutr. Diet..

[13] Santos H.O., Macedo R.C.O. (2018). Impact of intermittent fasting on the lipid profile: Assessment associated with diet and weight loss. Clin. Nutr. ESPEN.

[14] Mattson M.P., Longo V.D., Harvie M. (2017). Impact of intermittent fasting on health and disease processes. Ageing Res. Rev..

[15] Donahue S.W., Vaughan M.R., Demers L.M., Donahue H.J. (2003). Serum markers of bone metabolism show bone loss in hibernating bears. Clin. Orthop. Relat. Res..

[16] Hisatomi Y., Kugino K. (2019). Changes in bone density and bone quality caused by single fasting for 96 hours in rats. PeerJ.

[17] Alrowaili M.G., Hussein A.M., Eid E.A., Serria M.S., Abdellatif H., Sakr H.F. (2021). Effect of Intermittent Fasting on Glucose Homeostasis and Bone Remodeling in Glucocorticoid-Induced Osteoporosis Rat Model. J. Bone Metab..

[18] Xu X., Ding J., Wu X., Huang Z., Kong G., Liu Q., Yang Z., Huang Z., Zhu Q. (2019). Bone microstructure and metabolism changes under the combined intervention of ketogenic diet with intermittent fasting: An in vivo study of rats. Exp. Anim..

[19] Bahijri S.M., Ajabnoor G.M., Borai A., Al-Aama J.Y., Chrousos G.P. (2015). Effect of Ramadan fasting in Saudi Arabia on serum bone profile and immunoglobulins. Ther. Adv. Endocrinol. Metab..

[20] Kroll M.H. (2000). Parathyroid hormone temporal effects on bone formation and resorption. Bull. Math. Biol..

[21] Ben Nessib D., Maatallah K., Ferjani H., Kaffel D., Hamdi W. (2021). The potential effect of Ramadan fasting on musculoskeletal diseases: New perspectives. Clin. Rheumatol..

[22] Rodopaios N.E., Petridou A., Mougios V., Koulouri A.A., Vasara E., Papadopoulou S.K., Skepastianos P., Hassapidou M., Kafatos A.G. (2021). Vitamin D status, vitamin D intake, and sunlight exposure in adults adhering or not to periodic religious fasting for decades. Int. J. Food Sci. Nutr..

[23] Barnosky A., Kroeger C.M., Trepanowski J.F., Klempel M.C., Bhutani S., Hoddy K.K., Gabel K., Shapses S.A., Varady K.A. (2017). Effect of alternate day fasting on markers of bone metabolism: An exploratory analysis of a 6-month randomized controlled trial. Nutr. Healthy Aging.

[24] Clayton D.J., James L.J., Sale C., Templeman I., Betts J.A., Varley I. (2020). Severely restricting energy intake for 24 h does not affect markers of bone metabolism at rest or in response to re-feeding. Eur. J. Nutr..

[25] Martens C.R., Rossman M.J., Mazzo M.R., Jankowski L.R., Nagy E.E., Denman B.A., Richey J.J., Johnson S.A., Ziemba B.P., Wang Y. (2020). Short-term time-restricted feeding is safe and feasible in non-obese healthy midlife and older adults. Geroscience.

[26] Most J., Tosti V., Redman L.M., Fontana L. (2017). Calorie restriction in humans: An update. Ageing Res. Rev..

[27] Welton S., Minty R., O’Driscoll T., Willms H., Poirier D., Madden S., Kelly L. (2020). Intermittent fasting and weight loss: Systematic review. Can. Fam. Physician.

[28] Veronese N., Stubbs B., Koyanagi A., Hebert J.R., Cooper C., Caruso M.G., Guglielmi G., Reginster J.Y., Rizzoli R., Maggi S. (2018). Pro-inflammatory dietary pattern is associated with fractures in women: An eight-year longitudinal cohort study. Osteoporos. Int..

[29] Liang Y., Gao Y., Hua R., Lu M., Chen H., Wang Z., Li L., Hu K., Yin Y., Xu K. (2021). Calorie intake rather than food quantity consumed is the key factor for the anti-aging effect of calorie restriction. Aging.

[30] Cao J.J. (2018). Caloric restriction combined with exercise is effective in reducing adiposity and mitigating bone structural deterioration in obese rats. Ann. N. Y. Acad. Sci..

[31] McGrath C., Sankaran J.S., Misaghian-Xanthos N., Sen B., Xie Z., Styner M.A., Zong X., Rubin J., Styner M. (2020). Exercise Degrades Bone in Caloric Restriction, Despite Suppression of Marrow Adipose Tissue (MAT). J. Bone Miner Res..

[32] Devlin M.J., Cloutier A.M., Thomas N.A., Panus D.A., Lotinun S., Pinz I., Baron R., Rosen C.J., Bouxsein M.L. (2010). Caloric restriction leads to high marrow adiposity and low bone mass in growing mice. J. Bone Miner Res..

[33] Singh L., Tyagi S., Myers D., Duque G. (2018). Good, Bad, or Ugly: The Biological Roles of Bone Marrow Fat. Curr. Osteoporos. Rep..

[34] Duque G., Al Saedi A., Rivas D., Miard S., Ferland G., Picard F., Gaudreau P. (2020). Differential Effects of Long-Term Caloric Restriction and Dietary Protein Source on Bone and Marrow Fat of the Aging Rat. J. Gerontol. A Biol. Sci. Med. Sci..

[35] Devlin M.J., Brooks D.J., Conlon C., Vliet M., Louis L., Rosen C.J., Bouxsein M.L. (2016). Daily leptin blunts marrow fat but does not impact bone mass in calorie-restricted mice. J. Endocrinol..

[36] Talbott S.M., Cifuentes M., Dunn M.G., Shapses S.A. (2001). Energy restriction reduces bone density and biomechanical properties in aged female rats. J. Nutr..

[37] Bodnar M., Skalicky M., Viidik A., Erben R.G. (2012). Interaction between exercise, dietary restriction and age-related bone loss in a rodent model of male senile osteoporosis. Gerontology.

[38] LaMothe J.M., Hepple R.T., Zernicke R.F. (2003). Selected contribution: Bone adaptation with aging and long-term caloric restriction in Fischer 344 x Brown-Norway F1-hybrid rats. J. Appl. Physiol..

[39] Behrendt A.K., Kuhla A., Osterberg A., Polley C., Herlyn P., Fischer D.C., Scotland M., Wree A., Histing T., Menger M.D. (2016). Dietary Restriction-Induced Alterations in Bone Phenotype: Effects of Lifelong Versus Short-Term Caloric Restriction on Femoral and Vertebral Bone in C57BL/6 Mice. J. Bone Miner Res..

[40] Ahn H., Seo D.H., Kim H.S., Choue R. (2014). Calorie restriction aggravated cortical and trabecular bone architecture in ovariectomy-induced estrogen-deficient rats. Nutr. Res..

[41] Hamrick M.W., Ding K.H., Ponnala S., Ferrari S.L., Isales C.M. (2008). Caloric restriction decreases cortical bone mass but spares trabecular bone in the mouse skeleton: Implications for the regulation of bone mass by body weight. J. Bone Miner Res..

[42] Aikawa Y., Agata U., Kakutani Y., Kato S., Noma Y., Hattori S., Ogata H., Ezawa I., Omi N. (2016). The Preventive Effect of Calcium Supplementation on Weak Bones Caused by the Interaction of Exercise and Food Restriction in Young Female Rats During the Period from Acquiring Bone Mass to Maintaining Bone Mass. Calcif. Tissue Int..

[43] Jeon Y.K., Kim W.J., Shin M.J., Chung H.Y., Kim S.S., Kim B.H., Kim S.J., Kim Y.K., Kim I.J. (2014). Short-term caloric restriction does not reduce bone mineral density in rats with early type 2 diabetes. Endocrinol. Metab..

[44] Fontinele R.G., Krause Neto W., Gama E.F., Brito Mari R., de Souza R.R., Conrado A., Mochizuki L., Kfoury Junior J.R. (2017). Caloric restriction minimizes aging effects on the femoral medial condyle. Aging Male.

[45] Villareal D.T., Kotyk J.J., Armamento-Villareal R.C., Kenguva V., Seaman P., Shahar A., Wald M.J., Kleerekoper M., Fontana L. (2011). Reduced bone mineral density is not associated with significantly reduced bone quality in men and women practicing long-term calorie restriction with adequate nutrition. Aging Cell.

[46] Villareal D.T., Fontana L., Weiss E.P., Racette S.B., Steger-May K., Schechtman K.B., Klein S., Holloszy J.O. (2006). Bone mineral density response to caloric restriction-induced weight loss or exercise-induced weight loss: A randomized controlled trial. Arch. Intern. Med..

[47] Villareal D.T., Fontana L., Das S.K., Redman L., Smith S.R., Saltzman E., Bales C., Rochon J., Pieper C., Huang M. (2016). Effect of Two-Year Caloric Restriction on Bone Metabolism and Bone Mineral Density in Non-Obese Younger Adults: A Randomized Clinical Trial. J. Bone Miner Res..

[48] Razny U., Goralska J., Calder P.C., Gruca A., Childs C.E., Kapusta M., Slowinska-Solnica K., Dembinska-Kiec A., Solnica B., Malczewska-Malec M. (2021). The Effect of Caloric Restriction with and without n-3 PUFA Supplementation on Bone Turnover Markers in Blood of Subjects with Abdominal Obesity: A Randomized Placebo-Controlled Trial. Nutrients.

[49] Yasuda N., Yano T. (2018). Concomitant assessment of DNA oxidation and bone resorption over a rapid body mass reduction period in female judokas. J. Biol. Regul. Homeost. Agents.

[50] Villareal D.T., Chode S., Parimi N., Sinacore D.R., Hilton T., Armamento-Villareal R., Napoli N., Qualls C., Shah K. (2011). Weight loss, exercise, or both and physical function in obese older adults. N. Engl. J. Med..

[51] Flanagan E.W., Most J., Mey J.T., Redman L.M. (2020). Calorie Restriction and Aging in Humans. Annu. Rev. Nutr..

[52] Locher J.L., Goldsby T.U., Goss A.M., Kilgore M.L., Gower B., Ard J.D. (2016). Calorie restriction in overweight older adults: Do benefits exceed potential risks?. Exp. Gerontol..

[53] Hargreaves S.M., Raposo A., Saraiva A., Zandonadi R.P. (2021). Vegetarian Diet: An Overview through the Perspective of Quality of Life Domains. Int. J. Environ. Res. Public Health.

[54] Rizzo N.S., Sabate J., Jaceldo-Siegl K., Fraser G.E. (2011). Vegetarian dietary patterns are associated with a lower risk of metabolic syndrome: The adventist health study 2. Diabetes Care.

[55] Lau E.M., Kwok T., Woo J., Ho S.C. (1998). Bone mineral density in Chinese elderly female vegetarians, vegans, lacto-vegetarians and omnivores. Eur. J. Clin. Nutr..

[56] Ho-Pham L.T., Nguyen N.D., Nguyen T.V. (2009). Effect of vegetarian diets on bone mineral density: A Bayesian meta-analysis. Am. J. Clin. Nutr..

[57] Iguacel I., Miguel-Berges M.L., Gomez-Bruton A., Moreno L.A., Julian C. (2019). Veganism, vegetarianism, bone mineral density, and fracture risk: A systematic review and meta-analysis. Nutr. Rev..

[58] Pawlak R. (2021). Vitamin B12 status is a risk factor for bone fractures among vegans. Med. Hypotheses.

[59] Chuang T.L., Koo M., Chuang M.H., Lin C.H., Huang C.H., Wang Y.F. (2022). Changes in Bone Mineral Density and Trabecular Bone Score over Time between Vegetarian and Non-Vegetarian Middle-Aged and Older Women: A Three-Year Retrospective Medical Record Review. Int. J. Environ. Res. Public Health.

[60] Knurick J.R., Johnston C.S., Wherry S.J., Aguayo I. (2015). Comparison of correlates of bone mineral density in individuals adhering to lacto-ovo, vegan, or omnivore diets: A cross-sectional investigation. Nutrients.

[61] Tesar R., Notelovitz M., Shim E., Kauwell G., Brown J. (1992). Axial and peripheral bone density and nutrient intakes of postmenopausal vegetarian and omnivorous women. Am. J. Clin. Nutr..

[62] Chuang T.L., Lin C.H., Wang Y.F. (2021). Effects of vegetarian diet on bone mineral density. Tzu Chi Med. J..

[63] Brants H.A., Löwik M.R., Westenbrink S., Hulshof K.F., Kistemaker C. (1990). Adequacy of a vegetarian diet at old age (Dutch Nutrition Surveillance System). J. Am. Coll. Nutr..

[64] Berg J., Seyedsadjadi N., Grant R. (2020). Increased Consumption of Plant Foods Is Associated with Increased Bone Mineral Density. J. Nutr. Health Aging.

[65] Aleksandrova K., Koelman L., Rodrigues C.E. (2021). Dietary patterns and biomarkers of oxidative stress and inflammation: A systematic review of observational and intervention studies. Redox Biol..

[66] Sahni S., Hannan M.T., Blumberg J., Cupples L.A., Kiel D.P., Tucker K.L. (2009). Inverse association of carotenoid intakes with 4-y change in bone mineral density in elderly men and women: The Framingham Osteoporosis Study. Am. J. Clin. Nutr..

[67] Burckhardt P. (2016). The role of low acid load in vegetarian diet on bone health: A narrative review. Swiss Med. Wkly..

[68] Baker K.D., Loughman A., Spencer S.J., Reichelt A.C. (2017). The impact of obesity and hypercaloric diet consumption on anxiety and emotional behavior across the lifespan. Neurosci. Biobehav. Rev..

[69] Johnson R.J., Nakagawa T., Sanchez-Lozada L.G., Shafiu M., Sundaram S., Le M., Ishimoto T., Sautin Y.Y., Lanaspa M.A. (2013). Sugar, uric acid, and the etiology of diabetes and obesity. Diabetes.

[70] Kawano Y., Edwards M., Huang Y., Bilate A.M., Araujo L.P., Tanoue T., Atarashi K., Ladinsky M.S., Reiner S.L., Wang H.H. (2022). Microbiota imbalance induced by dietary sugar disrupts immune-mediated protection from metabolic syndrome. Cell.

[71] Frieler R.A., Vigil T.M., Song J., Leung C., Lumeng C.N., Mortensen R.M. (2021). High-fat and high-sodium diet induces metabolic dysfunction in the absence of obesity. Obesity.

[72] Atak S., Boye A., Peciña S., Liu Z.X. (2023). High-fat-sugar diet is associated with impaired hippocampus-dependent memory in humans. Physiol. Behav..

[73] Tsanzi E., Fitch C.W., Tou J.C. (2008). Effect of consuming different caloric sweeteners on bone health and possible mechanisms. Nutr. Rev..

[74] Ahn H., Park Y.K. (2021). Sugar-sweetened beverage consumption and bone health: A systematic review and meta-analysis. Nutr. J..

[75] Bragança M., Bogea E.G., de Almeida Fonseca Viola P.C., Dos Santos Vaz J., Confortin S.C., Menezes A.M.B., Gonçalves H., Bettiol H., Barbieri M.A., Cardoso V.C. (2023). High Consumption of Sugar-Sweetened Beverages Is Associated with Low Bone Mineral Density in Young People: The Brazilian Birth Cohort Consortium. Nutrients.

[76] Bennett A.M., Murray K., Ambrosini G.L., Oddy W.H., Walsh J.P., Zhu K. (2022). Prospective Associations of Sugar-Sweetened Beverage Consumption During Adolescence with Body Composition and Bone Mass at Early Adulthood. J. Nutr..

[77] Tjäderhane L., Larmas M. (1998). A high sucrose diet decreases the mechanical strength of bones in growing rats. J. Nutr..

[78] Bass E.F., Baile C.A., Lewis R.D., Giraudo S.Q. (2013). Bone quality and strength are greater in growing male rats fed fructose compared with glucose. Nutr. Res..

[79] Tsanzi E., Light H.R., Tou J.C. (2008). The effect of feeding different sugar-sweetened beverages to growing female Sprague-Dawley rats on bone mass and strength. Bone.

[80] Felice J.I., Gangoiti M.V., Molinuevo M.S., McCarthy A.D., Cortizo A.M. (2014). Effects of a metabolic syndrome induced by a fructose-rich diet on bone metabolism in rats. Metabolism.

[81] Minematsu A., Nishii Y., Sakata S. (2018). High-fat/high-sucrose diet results in higher bone mass in aged rats. Bone Rep..

[82] Vandewalle S., Taes Y., Van Helvoirt M., Debode P., Herregods N., Ernst C., Roef G., Van Caenegem E., Roggen I., Verhelle F. (2013). Bone size and bone strength are increased in obese male adolescents. J. Clin. Endocrinol. Metab..

[83] Kim H.Y., Jung H.W., Hong H., Kim J.H., Shin C.H., Yang S.W., Lee Y.A. (2017). The Role of Overweight and Obesity on Bone Health in Korean Adolescents with a Focus on Lean and Fat Mass. J. Korean Med. Sci..

[84] Leonard M.B., Zemel B.S., Wrotniak B.H., Klieger S.B., Shults J., Stallings V.A., Stettler N. (2015). Tibia and radius bone geometry and volumetric density in obese compared to non-obese adolescents. Bone.

[85] Woo D.G., Lee B.Y., Lim D., Kim H.S. (2009). Relationship between nutrition factors and osteopenia: Effects of experimental diets on immature bone quality. J. Biomech..

[86] Inzana J.A., Kung M., Shu L., Hamada D., Xing L.P., Zuscik M.J., Awad H.A., Mooney R.A. (2013). Immature mice are more susceptible to the detrimental effects of high fat diet on cancellous bone in the distal femur. Bone.

[87] Wee N.K.Y., Enriquez R.F., Nguyen A.D., Horsnell H., Kulkarni R., Khor E.C., Herzog H., Baldock P.A. (2018). Diet-induced obesity suppresses cortical bone accrual by a neuropeptide Y-dependent mechanism. Int. J. Obes..

[88] Tang L., Yang X., Gao X., Du H., Han Y., Zhang D., Wang Z., Sun L. (2016). Inhibiting myostatin signaling prevents femoral trabecular bone loss and microarchitecture deterioration in diet-induced obese rats. Exp. Biol. Med..

[89] Zhang Z., Zhang Z., Pei L., Zhang X., Li B., Meng Y., Zhou X. (2022). How high-fat diet affects bone in mice: A systematic review and meta-analysis. Obes. Rev..

[90] Doucette C.R., Horowitz M.C., Berry R., MacDougald O.A., Anunciado-Koza R., Koza R.A., Rosen C.J. (2015). A High Fat Diet Increases Bone Marrow Adipose Tissue (MAT) But Does Not Alter Trabecular or Cortical Bone Mass in C57BL/6J Mice. J. Cell Physiol..

[91] Liu S.Q., Wu J., Mo J., Sun Z.X., Yang H.B., Huang C.W., Lei M.X., Peng L.W., Xu L. (2009). Serum leptin level and its association with bone mineral density in obese children. Zhongguo Dang Dai Er Ke Za Zhi.

[92] Głogowska-Szeląg J., Kos-Kudła B., Marek B., Nowak M., Siemińska L. (2019). Assessment of selected adipocytokines in obese women with postmenopausal osteoporosis. Endokrynol. Pol..

[93] Ducy P., Amling M., Takeda S., Priemel M., Schilling A.F., Beil F.T., Shen J., Vinson C., Rueger J.M., Karsenty G. (2000). Leptin inhibits bone formation through a hypothalamic relay: A central control of bone mass. Cell.

[94] López-Gómez J.J., Pérez-Castrillón J.L., García de Santos I., Pérez-Alonso M., Izaola-Jauregui O., Primo-Martín D., De Luis-Román D.A. (2022). Influence of Obesity on Bone Turnover Markers and Fracture Risk in Postmenopausal Women. Nutrients.

[95] Kim S.H., Lee J.W., Im J.A., Hwang H.J. (2010). Serum osteocalcin is related to abdominal obesity in Korean obese and overweight men. Clin. Chim. Acta.

[96] Wang T., He C. (2018). Pro-inflammatory cytokines: The link between obesity and osteoarthritis. Cytokine Growth Factor Rev..

[97] Abuna R.P., De Oliveira F.S., Santos Tde S., Guerra T.R., Rosa A.L., Beloti M.M. (2016). Participation of TNF-α in Inhibitory Effects of Adipocytes on Osteoblast Differentiation. J. Cell Physiol..

[98] Vranić L., Mikolašević I., Milić S. (2019). Vitamin D Deficiency: Consequence or Cause of Obesity?. Medicina.

[99] Patsch J.M., Kiefer F.W., Varga P., Pail P., Rauner M., Stupphann D., Resch H., Moser D., Zysset P.K., Stulnig T.M. (2011). Increased bone resorption and impaired bone microarchitecture in short-term and extended high-fat diet-induced obesity. Metabolism.

[100] Ootsuka T., Nakanishi A., Tsukamoto I. (2015). Increase in osteoclastogenesis in an obese Otsuka Long-Evans Tokushima fatty rat model. Mol Med Rep..

[101] Han J., Wei L., Xu W., Lu J., Wang C., Bao Y., Jia W. (2015). CTSK inhibitor exert its anti-obesity effects through regulating adipocyte differentiation in high-fat diet induced obese mice. Endocr. J..

[102] Shu L., Beier E., Sheu T., Zhang H., Zuscik M.J., Puzas E.J., Boyce B.F., Mooney R.A., Xing L. (2015). High-fat diet causes bone loss in young mice by promoting osteoclastogenesis through alteration of the bone marrow environment. Calcif. Tissue Int..

[103] Oshima K., Nampei A., Matsuda M., Iwaki M., Fukuhara A., Hashimoto J., Yoshikawa H., Shimomura I. (2005). Adiponectin increases bone mass by suppressing osteoclast and activating osteoblast. Biochem. Biophys. Res. Commun..

[104] Wang Q.P., Li X.P., Wang M., Zhao L.L., Li H., Xie H., Lu Z.Y. (2014). Adiponectin exerts its negative effect on bone metabolism via OPG/RANKL pathway: An in vivo study. Endocrine.

[105] Montalvany-Antonucci C.C., Zicker M.C., Ferreira A.V.M., Macari S., Ramos-Junior E.S., Gomez R.S., Pereira T.S.F., Madeira M.F.M., Fukada S.Y., Andrade I. (2018). High-fat diet disrupts bone remodeling by inducing local and systemic alterations. J. Nutr. Biochem..

[106] Li W., Xu P., Wang C., Ha X., Gu Y., Wang Y., Zhang J., Xie J. (2017). The effects of fat-induced obesity on bone metabolism in rats. Obes. Res. Clin. Pract..

[107] Cai F., Yusufu A., Liu K., Chen W., Zhao R., Liu Y., Liu Y. (2023). High-fat diet causes undesirable bone regeneration by altering the bone marrow environment in rats. Front. Endocrinol..

[108] Cortez M., Carmo L.S., Rogero M.M., Borelli P., Fock R.A. (2013). A high-fat diet increases IL-1, IL-6, and TNF-α production by increasing NF-κB and attenuating PPAR-γ expression in bone marrow mesenchymal stem cells. Inflammation.

[109] Steppan C.M., Lazar M.A. (2002). Resistin and obesity-associated insulin resistance. Trends Endocrinol. Metab..

[110] De Benedetti F., Rucci N., Del Fattore A., Peruzzi B., Paro R., Longo M., Vivarelli M., Muratori F., Berni S., Ballanti P. (2006). Impaired skeletal development in interleukin-6-transgenic mice: A model for the impact of chronic inflammation on the growing skeletal system. Arthritis Rheum..

[111] Xiao Y., Cui J., Li Y.X., Shi Y.H., Wang B., Le G.W., Wang Z.P. (2011). Dyslipidemic high-fat diet affects adversely bone metabolism in mice associated with impaired antioxidant capacity. Nutrition.

[112] Zengin A., Kropp B., Chevalier Y., Junnila R., Sustarsic E., Herbach N., Fanelli F., Mezzullo M., Milz S., Bidlingmaier M. (2016). Low-carbohydrate, high-fat diets have sex-specific effects on bone health in rats. Eur. J. Nutr..

[113] Dong X.L., Li C.M., Cao S.S., Zhou L.P., Wong M.S. (2016). A High-Saturated-Fat, High-Sucrose Diet Aggravates Bone Loss in Ovariectomized Female Rats. J. Nutr..

[114] Zernicke R.F., Salem G.J., Barnard R.J., Schramm E. (1995). Long-term, high-fat-sucrose diet alters rat femoral neck and vertebral morphology, bone mineral content, and mechanical properties. Bone.

[115] Tian L., Wang C., Xie Y., Wan S., Zhang K., Yu X. (2018). High Fructose and High Fat Exert Different Effects on Changes in Trabecular Bone Micro-structure. J. Nutr. Health Aging.

[116] Jatkar A., Kurland I.J., Judex S. (2017). Diets High in Fat or Fructose Differentially Modulate Bone Health and Lipid Metabolism. Calcif. Tissue Int..

[117] Lorincz C., Reimer R.A., Boyd S.K., Zernicke R.F. (2010). High-fat, sucrose diet impairs geometrical and mechanical properties of cortical bone in mice. Br. J. Nutr..

[118] Li K.C., Zernicke R.F., Barnard R.J., Li A.F. (1990). Effects of a high fat-sucrose diet on cortical bone morphology and biomechanics. Calcif. Tissue Int..

[119] World Health Organization (2007). Protein and Amino Acid Requirements in Human Nutrition.

[120] Cao J.J. (2017). High Dietary Protein Intake and Protein-Related Acid Load on Bone Health. Curr. Osteoporos. Rep..

[121] Shams-White M.M., Chung M., Du M., Fu Z., Insogna K.L., Karlsen M.C., LeBoff M.S., Shapses S.A., Sackey J., Wallace T.C. (2017). Dietary protein and bone health: A systematic review and meta-analysis from the National Osteoporosis Foundation. Am. J. Clin. Nutr..

[122] Antonio J., Ellerbroek A., Evans C., Silver T., Peacock C.A. (2018). High protein consumption in trained women: Bad to the bone?. J. Int. Soc. Sports Nutr..

[123] Calvez J., Poupin N., Chesneau C., Lassale C., Tomé D. (2012). Protein intake, calcium balance and health consequences. Eur. J. Clin. Nutr..

[124] Kerstetter J.E., Caseria D.M., Mitnick M.E., Ellison A.F., Gay L.F., Liskov T.A., Carpenter T.O., Insogna K.L. (1997). Increased circulating concentrations of parathyroid hormone in healthy, young women consuming a protein-restricted diet. Am. J. Clin. Nutr..

[125] Langdahl B.L., Kassem M., Møller M.K., Eriksen E.F. (1998). The effects of IGF-I and IGF-II on proliferation and differentiation of human osteoblasts and interactions with growth hormone. Eur. J. Clin. Investig..

[126] Dawson-Hughes B., Harris S.S., Rasmussen H., Song L., Dallal G.E. (2004). Effect of dietary protein supplements on calcium excretion in healthy older men and women. J. Clin. Endocrinol. Metab..

[127] Tirapegui J., Ribeiro S.M., Pires I.S., Rogero M.M. (2012). Effects of two different levels of dietary protein on body composition and protein nutritional status of growing rats. Nutrients.

[128] Nebot E., Aparicio V.A., Coll-Risco I., Camiletti-Moirón D., Schneider J., Kapravelou G., Heimel P., Martínez R., Andrade A., Slezak P. (2016). Effects of a moderately high-protein diet and interval aerobic training combined with strength-endurance exercise on markers of bone metabolism, microarchitecture and turnover in obese Zucker rats. Bone.

[129] Kerstetter J.E., O’Brien K.O., Insogna K.L. (2003). Dietary protein, calcium metabolism, and skeletal homeostasis revisited. Am. J. Clin. Nutr..

[130] Matikainen N., Pekkarinen T., Ryhänen E.M., Schalin-Jäntti C. (2021). Physiology of Calcium Homeostasis: An Overview. Endocrinol. Metab. Clin. N. Am..

[131] Yao X., Hu J., Kong X., Zhu Z. (2021). Association between Dietary Calcium Intake and Bone Mineral Density in Older Adults. Ecol. Food Nutr..

[132] Closa-Monasterolo R., Zaragoza-Jordana M., Ferré N., Luque V., Grote V., Koletzko B., Verduci E., Vecchi F., Escribano J. (2018). Adequate calcium intake during long periods improves bone mineral density in healthy children. Data from the Childhood Obesity Project. Clin. Nutr..

[133] Ma X.M., Huang Z.W., Yang X.G., Su Y.X. (2014). Calcium supplementation and bone mineral accretion in Chinese adolescents aged 12–14 years: A 12-month, dose-response, randomised intervention trial. Br. J. Nutr..

[134] Vannucci L., Fossi C., Quattrini S., Guasti L., Pampaloni B., Gronchi G., Giusti F., Romagnoli C., Cianferotti L., Marcucci G. (2018). Calcium Intake in Bone Health: A Focus on Calcium-Rich Mineral Waters. Nutrients.

[135] Lee A.M., Sawyer R.K., Moore A.J., Morris H.A., O’Loughlin P.D., Anderson P.H. (2014). Adequate dietary vitamin D and calcium are both required to reduce bone turnover and increased bone mineral volume. J. Steroid Biochem. Mol. Biol..

[136] Carmeliet G., Dermauw V., Bouillon R. (2015). Vitamin D signaling in calcium and bone homeostasis: A delicate balance. Best Pract. Res. Clin. Endocrinol. Metab..

[137] Liu C., Kuang X., Li K., Guo X., Deng Q., Li D. (2020). Effects of combined calcium and vitamin D supplementation on osteoporosis in postmenopausal women: A systematic review and meta-analysis of randomized controlled trials. Food Funct..

[138] Bolland M.J., Leung W., Tai V., Bastin S., Gamble G.D., Grey A., Reid I.R. (2015). Calcium intake and risk of fracture: Systematic review. BMJ.

[139] Rizzoli R. (2022). Dairy products and bone health. Aging Clin. Exp. Res..

[140] Ratajczak A.E., Zawada A., Rychter A.M., Dobrowolska A., Krela-Kaźmierczak I. (2021). Milk and Dairy Products: Good or Bad for Human Bone? Practical Dietary Recommendations for the Prevention and Management of Osteoporosis. Nutrients.

[141] van den Heuvel E., Steijns J. (2018). Dairy products and bone health: How strong is the scientific evidence?. Nutr. Res. Rev..

[142] Li B.Y., Mahe J.L., Hao J.Y., Ye W.H., Bai X.F., Feng H.T., Szeto I.M., Jing L.P., Zhao Z.F., Chen Y.M. (2023). Formula Milk Supplementation and Bone Acquisition in 4-6 Years Chinese Children: A 12-Month Cluster-Randomized Controlled Trial. Nutrients.

[143] Gui J.C., Brašić J.R., Liu X.D., Gong G.Y., Zhang G.M., Liu C.J., Gao G.Q. (2012). Bone mineral density in postmenopausal Chinese women treated with calcium fortification in soymilk and cow’s milk. Osteoporos. Int..

[144] Michaëlsson K., Wolk A., Langenskiöld S., Basu S., Warensjö Lemming E., Melhus H., Byberg L. (2014). Milk intake and risk of mortality and fractures in women and men: Cohort studies. BMJ.

[145] Salari N., Darvishi N., Bartina Y., Larti M., Kiaei A., Hemmati M., Shohaimi S., Mohammadi M. (2021). Global prevalence of osteoporosis among the world older adults: A comprehensive systematic review and meta-analysis. J. Orthop. Surg. Res..

[146] Dai Z., Butler L.M., van Dam R.M., Ang L.W., Yuan J.M., Koh W.P. (2014). Adherence to a vegetable-fruit-soy dietary pattern or the Alternative Healthy Eating Index is associated with lower hip fracture risk among Singapore Chinese. J. Nutr..

